# Analyzing Discussions Around Rural Health on Twitter During the COVID-19 Pandemic: Social Network Analysis of Twitter Data

**DOI:** 10.2196/39209

**Published:** 2023-03-08

**Authors:** Wasim Ahmed, Josep Vidal-Alaball, Josep Maria Vilaseca Llobet

**Affiliations:** 1 Stirling University Management School University of Stirling Stirling United Kingdom; 2 Unitat de Suport a la Recerca de la Catalunya Central Fundació Institut Universitari per a la Recerca a l’Atenció Primària de Salut Jordi Gol i Gurina Sant Fruitós de Bages Spain; 3 Health Promotion in Rural Areas Research Group Gerència Territorial de la Catalunya Central Institut Català de la Salut Sant Fruitós de Bages Spain; 4 Faculty of Medicine University of Vic – Central University of Catalonia Vic Spain; 5 Primary Care Service Althaia Xarxa Assistencial Universitària de Manresa Manresa Spain

**Keywords:** rural health, Twitter messaging, social media, COVID-19, SARS-CoV-2, coronavirus, social network analysis

## Abstract

**Background:**

Individuals from rural areas are increasingly using social media as a means of communication, receiving information, or actively complaining of inequalities and injustices.

**Objective:**

The aim of our study is to analyze conversations about rural health taking place on Twitter during a particular phase of the COVID-19 pandemic.

**Methods:**

This study captured 57 days’ worth of Twitter data related to rural health from June to August 2021, using English-language keywords. The study used social network analysis and natural language processing to analyze the data.

**Results:**

It was found that Twitter served as a fruitful platform to raise awareness of problems faced by users living in rural areas. Overall, Twitter was used in rural areas to express complaints, debate, and share information.

**Conclusions:**

Twitter could be leveraged as a powerful social listening tool for individuals and organizations that want to gain insight into popular narratives around rural health.

## Introduction

Globalization and proliferation of the world wide web and social media have increased the amount of information available internationally. Access to information can be crucial in rural areas as it can help break the traditional isolation that those living in rural areas experience. In the past, it was believed that rural communities were isolated, with poor access to web-based information and being excluded from social media. This is partially true [1]. However, in recent times, in both high-income and transitional countries, a remarkable number of individuals from rural areas are using social media to communicate to receive up-to-date information and access quality health support and services [2].

It is well known that several societal and health issues are unique to rural areas when compared to those in urban areas. This includes high poverty rates, less access to health care, a higher percentage of adults with health problems, and health issues related to exposure to chemicals used in farming.

Twitter is a popular form of social media, and its use by health care professionals has been studied extensively [3]. Some examples include the use of Twitter as a means of health promotion by large urban hospitals and clinics in the United States [4]. Moreover, Twitter has also been used as a new source of data to study depression and its wider determinants in deprived populations in India and Brazil and for predictive analytics and sentiment analysis [5].

A recent study analyzing the implications of Twitter in health-related research identified a wide variety of themes ranging from professional education in health care to big data, social marketing and substance use, physical and emotional well-being of young adults, and public health and health communication [6]. The analysis of social media provides a useful tool for public health specialists and government decision-makers to gain insight into population reactions and feelings [7], especially in times of uncertainty such as the one we are facing with the present pandemic [8].

Misinformation has been a problem on social media platforms such as Twitter. A systematic review of the prevalence of health misinformation on social media before the COVID-19 pandemic found that 2 of the 6 principal categories were vaccines (32%) and pandemics (10%). The prevalence of health misinformation was the highest on Twitter [9]. Another paper published after the onset of the COVID-19 pandemic suggests understudied research areas that need to be addressed to improve policy and practice in response to health misinformation; those research areas include (1) spatial, temporal, network, and cross-platform dynamics of misinformation sharing and (2) the focus on vulnerable populations [10]. A recent bibliometric study of the scientific literature on medical and health-related misinformation on social media found that the most popularly investigated social media platform is Twitter and that COVID-19 is a common topic investigated across all platforms [11].

A study by Cuomo et al [12] analyzed the geospatial distribution of Tweets related to COVID-19 to try to illustrate the full scope of the pandemic. The authors found that rural areas in the United States engaged in COVID-19–related social media conversations at later stages of the outbreak than urban areas [12]. A person’s birthplace has been regarded as an important determinant of health [13]. The availability of resources in rural areas differs from that in urban areas, and this has an impact on population health [14,15]. Another problem in rural areas is the shortage of health professionals willing to work in these areas [16]. Some initiatives are being developed to promote interest in rural health in this context. One such initiative uses social media for this objective. This is the case of the Rural Family Medicine Café, which, since 2015, has been organizing regular meetings using social networks to put in contact health professionals who work or have an interest in rural health [17,18].

There are few studies investigating the use of Twitter in relation to rural health issues that analyze popular topics covered in these areas. This is particularly interesting at the time of the COVID-19 pandemic, as contrary to the initial beliefs that lower population density could protect against the virus, COVID-19 did not spare these areas [19].

The main overall aim of our study is to analyze the conversations related to rural health taking place on Twitter during the COVID-19 pandemic to better understand popular narratives being communicated. Twitter is a popular social networking platform, and our study aims to shed light on the content hosted on the platform related to rural health.

More specifically, the objectives of this study are to study a particular time frame to (1) develop an understanding of the content and debates being shared on Twitter related to rural health, (2) to identify influential users around rural health on Twitter, and (3) uncover the key hashtags and websites being shared.

By fulfilling these objectives, the study will gain an understanding of rural health conversations taking place on Twitter during a specific phase of the COVID-19 pandemic between June and August 2021. The results are likely to be of interest to other scholars working in these areas as well as public health organizations and activists.

## Methods

### Sampling Tweets

This study made use of the Twitter Archiving Google Sheets (TAGS) tool to retrieve 15,586 tweets matching the keyword “rural health.” TAGS draws upon the Twitter Search application programming interface to retrieve tweets. Although an English keyword is used, other languages may also exist, for example, if they reply to a tweet using a different language but the original tweet was posted in English or if they quote or reply to a tweet. Tweets were retrieved from June 10 to August 6, 2021, encompassing 57 days during the COVID-19 pandemic. No particular geographical location was selected from which to retrieve tweets, and tweets could be sent and received from anywhere in the world where Twitter is available. This is not a limitation of this study per se*,* as Twitter does not provide accurate location-based data and many studies are conducted using keywords. It is important to mention that there are numerous definitions of “rural” [20]. These definitions differ in the cutoff point. To avoid disputes, we use the simple principle that if one thinks one is rural, one probably is.

Although it can be argued that tweets are in the public domain, the project was careful not to draw attention to individual users acting in a personal capacity (preventing unwanted exposure). However, the users and key tweets reproduced in this study derive either from accounts and users in the public domain, social media influencers, health organizations, politicians, and academic journals.

### Data Analysis

In order to identify influential users, the metric of betweenness centrality (the influence a user exerts on other users by his/her tweets) was applied, which is derived from the network theory and has been used in this study to find Twitter users that have an influence in our data set. This methodology has been used in previous research [21-23]. This metric was used in this study as it can identify users located in strategic locations within the network and who are gatekeepers of information propagation. It is commonly used in social media research to find important users in a network. The betweenness centrality scores are unique to each network and can be used to benchmark each of the users and rank them from most to least influential by betweenness centrality. Providing a detailed overview of network visualizations is beyond the scope of this study. Those new to network visualizations may wish to examine research in this area, which outlines common network patterns and how to interpret them [24].

### Social Network Analysis

The software NodeXL (Social Media Research Foundation) was used to conduct a social network analysis of the data [17]. The network graph was laid out using the Clauset-Newman-Moore layout algorithm that is integrated into NodeXL. Social network analysis is the process of investigating social structures using networks and graph theory. This entails identifying and analyzing relations among entities and features in a social system. In our case, we analyzed the relationship among users by examining interaction patterns (retweets, replies, mentions, etc). We examined the whole network without prefiltering.

### Time-Series Analysis

Time-series analysis is the study of data over time (Multimedia Appendix 1); it is the process of examining a time series to understand it and make predictions about future trends based on past data. It has applications in many areas, including economics, biology and medicine, engineering, environmental science, and meteorology. In this study, we made use of time-series analysis to gain an understanding of the volume of all tweet types across time.

### Content Analysis and Natural Language Processing

NodeXL was also used to identify co-occurring word pairs, which is a type of natural language processing modality. In a word pair analysis, we looked at all the words in our data set and compared them to each other using their co-occurrence statistics (Multimedia Appendix 2). We then used these statistics to find all the word pairs that are likely to occur together more often than expected by chance.

### Ethics Approval

The study received ethical approval from Newcastle University (26055/2022).

## Results

### Results of Social Network Analysis

**Figure 1:**
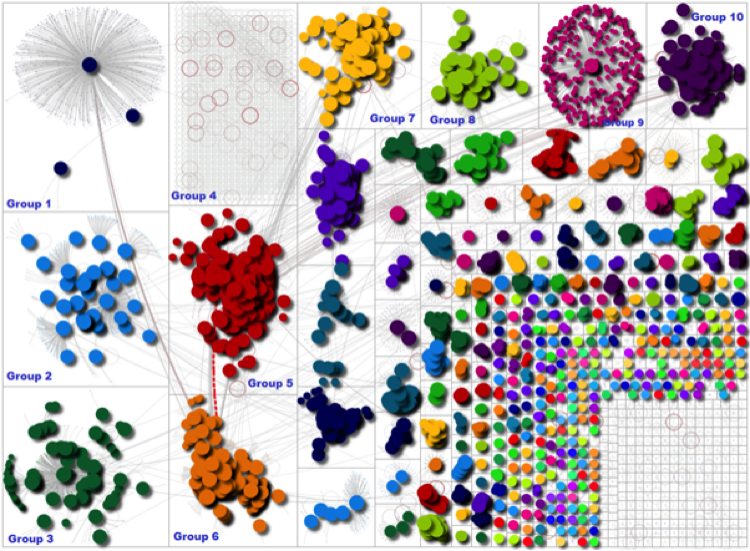
Social network visualization and discussions across groups.

Figure 1 provides a visual representation of Twitter activity based on the data that were captured. The circles within the network represent individual Twitter users who were tweeting using the words “rural health,” and the lines between them represent connections such as mentions and replies. Different colors are used to distinguish each of the groups, and they are listed from left to right, ranked by size, where group 1 is the largest cluster in the network, followed by group 2, and so forth.

The figure highlights how there were several different groups of users who were conversing about different topics related to rural health. The largest group in the network (group 1) is that of a broadcast network where a user’s tweet is retweeted with high frequency.

There are also several other smaller groups and broadcast networks providing the overall network a community shape. Other popular groups, such as groups 3, 5, 6, 7, and 10, indicate that there were different communities forming on Twitter, which were conversing about different topics related to rural health. There is little engagement (retweets and mentions) among users within some groups, but cross-group interaction was seen to occur between groups 5 and 9. Groups 1 and 9 appeared to be broadcast as network shapes. Multimedia Appendix 2 contains a full list of keywords associated with each of the clusters, providing insight into the types of topics that were being discussed.

More specifically, to provide more context on the content of the groups and communities represented within the visual, a range of news articles and reports were being amplified. For example, one article shared in group one was entitled, “India’s healthcare workers are busting misinformation on WhatsApp.” This was the most dominant news story being amplified in group 1. Aside from “rural, health” itself, the most popular word pair in group 1 was that of “fighting, covid” (n=873). Other interesting keywords identified within this group included “busting, myths” (n=873), indicating the combatting of misinformation, which was also linked to the aforementioned news article. If we cross-reference to the top 10 retweets and examine the tweet ranked as the third-most popular, it can be seen that this tweet uses the keyword “busting myths.” In group 2, interesting word combinations (provided within the appendix) included “health, systems” (n=373), “expanding, medicaid” (n=256), “taxpayers, money” (n=254), and “affordable, health” (n=212). These keywords provide insight into the commonly used words and are helpful in understanding some of the topics that users were discussing. Multimedia Appendix 2 provides insight into groups 3 to 10 and an insight into some of the topics discussed.

### Results of the Time-Series Analysis

Multimedia Appendix 1 provides an overview of the data set’s unique edges (ie, tweets, retweets, mentions, etc). There appears to be a constant stream of Twitter activity, with 2 large peaks observed on June 18 and July 16, 2021, respectively. Overall, there appears to be much more activity taking place during June 2021. Upon investigating the peaks that were occurring within the data, it was found that these peaks tended to relate to spikes in retweets due to the tweets contained among the top 10.

### Results Related to Key Users, Websites, Hashtags, and Retweets

Table 1 provides insight into the key users. The first key user is the account of Akhilesh Yadav, a socialist leader of India. This is followed by the Twitter account of the World Health Organization and the Rural and Remote Health Journal Twitter, an open-access academic international journal. In fourth place, we found the account of Senator Reverend Raphael Warnock, a US senator from Georgia, and in fifth place, the account of the National Rural Health Association, a US nonprofit organization with the mission to provide leadership on rural health issues through advocacy, communications, education, and research.

Table 2 provides information about the top websites used in tweets. The top website used in tweets and by far (877 occurrences) is from The Verge, an American technology news website. It features an article on how health care workers are combating misinformation about COVID-19 in rural India. The second-most used website in tweets is also related to India and is based on an article from The New York Times, which describes how the bodies floating at the river Ganges were buried at their shores and showed that the authorities were not telling the truth about the full extent of the death toll caused by COVID-19. The third-most used website was from a suspended account and no longer accessible. Finally, the fourth-most used website was from Gary Votour, who was running for the post of governor of South Carolina, and the fifth-most used website was an article from IndiaSpend, an Indian web-based journal, which discussed how Indian rural health centers were struggling with staff shortages, especially pharmacists and doctors.

Table 3 provides insight into the top hashtags used in the tweets. The most used hashtag is #appoint_pharmacist_for_rural_health, a hashtag used in a campaign to advocate for the appointment of pharmacists in rural India. The second-most used hashtag was #33yearsofpmk, a hashtag commemorating 33 years of the Paattali Makkal Katchi (working people’s party), abbreviated as “PMK”—a political party in Tamil Nadu, India. The third-most used hashtag is directly related to rural health (#ruralhealth), and the fourth- and fifth-most used hashtags are 2 related hashtags, one in English and the other in Korean, to celebrate the birthday of Sunoo (birth name: Kim Sun-oo), a member of the Korean band ENHYPEN. This appears within the data because as result of the birthday packed lunches were delivered to the front-liners of the Los Banos, Laguna Rural Health Unit. The hashtags related to COVID-19 come in the 6th and 10th positions.

We also examined the top 10 retweets. It was found that the first, second, and fourth-ranking retweets were addressed to specific individuals. The most popular retweet was an appeal to the prime minister of India to appoint more rural doctors, and the second- and fourth-ranking retweets were related to a campaign to uncover water corruption in rural areas. The third-ranking retweet is a recognition of rural health activists who combat misinformation about COVID-19 in rural India. The other popular retweets had several purposes related to rural and public health: to report corruption related to rural health problems and the deplorable conditions of rural health care facilities, to congratulate a doctor by providing some key indicators of a rural health program milestone, to announce the building of health care facilities, and to report the shortage of health workforce and encourage professionals to work in rural areas.

**Table 1:** Key users by betweenness centrality.

User handle	Bio	Betweenness centrality
yadavakhilesh	Socialist Leader of India. Chief Minister of UP (2012 - 2017)	5185471
who	We are the #UnitedNations’ health agency - #HealthForAll. Always check our latest tweets on #COVID19 for updated advice/information.	5150502
rrh_journal	Open-access, peer-reviewed journal providing an international evidence base to inform improvement in rural and remote health (free-to-read, no page charges)	4270264
senatorwarnock	United States Senator from Georgia. Pastor of Ebenezer Baptist Church.	3855435
ruralhealth	National Rural Health Association, 21k+ members nationwide, providing leadership and support at NRHA.	3000683
bprophetable	Only way to get good politicians is get rid of bad ones. I try to retweet facts and everyone’s opinions including those I disagree with #FactsMatter	2476886
dainikbhaskar	India's Biggest Hindi Newspaper & News App. For Realtime News Updates, Local News for 2000 cities, Short Video News, Download our App: http://dainik-b.in/riOAhsOKg6	2403907
nytopinion	We amplify voices on the issues that matter to you. | Tell us what you think: letters@nytimes.com	2372269
timryan	Proud dad and husband, Ohio native, die-hard Browns fan. Running for U.S. Senate to fight like hell to cut workers in on the deal.	2349314
ruraldoctorsaus	Rural Doctors Association of Australia - promoting excellent medical care for rural and remote Australians.	2209906

**Table 2:** Top websites used in tweets.

Rank	Title	Tweets, n
1	India’s Healthcare Workers Are Busting Misinformation On WhatsApp	877
2	The Ganges Is Returning the Dead. It Does Not Lie.	164
3	This tweet is from a suspended account	115
4	Official campaign website for Gary Votour for Governor of South Carolina	89
5	As Third Wave Looms, Rural Health Centres Struggle With Expired Drugs, Missing Doctors	69
6	Myth Vs Facts Government of India has been working towards effective COVID-19 management in rural India by sustained strengthening of the Rural health Infrastructure, and through focussed Public Health Measures in active collaboration with the States	61
7	Gary Votour for South Carolina Governor campaign	60
8	Chhattisgarh to privatise rural health infra; public health experts and activists demand roll back	55
9	Official Account Of Chhattisgarh Pradesh Congress Committee.	47
10	Barak Obama's twitter account, it reads: Today, the Supreme Court upheld the Affordable Care Act. Again. This ruling reaffirms what we have long known to be true: the Affordable Care Act is here to stay.	47

**Table 3:** Top hashtags used in tweets.

Rank	Top hashtags
1	appoint_pharmacist_for_rural_health
2	33yearsofpmk
3	ruralhealth
4	sunooourmiracleofjune
5	﻿눈부신_선우의_열아홉번째_생일
6	covid19
7	pharmacistfederation
8	rural
9	medicaidsaveslivesact
10	covid

### Results Related to Language and Geographical Locations

On examining the retweet count, the most widely used language was English, either in non-English native-speaking areas or in countries where other local languages are spoken. This is likely to be influenced by the keywords that were used to retrieve data as these were in English. In total, 4553 (80%) retweets are written only in English. Including tweets that mix English and other languages accounts for 94.5% (n=5350) of retweets. The second-most used language is Tagalog, mixed with English in the main body of the Tweet (n=407, 7 %). The third-most used language is Korean, but in this case used only as a hashtag; the retweets’ main text is in English (n=390, 6.9%). The fourth-most used language is Hindi, only used in one of the top 10 retweets in our data set (n=311, 5.5%). Other languages can easily appear even if English keywords are used to retrieve the data because a tweet written in English can be quoted by a user writing in a different language, which would be included in our data set. The geographical locations of the debates are mainly India, Pakistan, Australia, the Philippines, and the United States. The most widely used language was English. Other languages used were Hindi, Korean, and Tagalog.

## Discussion

### Principal Findings

Although the role of social media in rural settings has been studied previously [2,18], to our knowledge, this is the first study on the specific use of Twitter in relation to rural health issues and has identified the common topics discussed in these settings at a specific point in time.

Our study also found that the key users related to this topic are individuals (mainly politicians) and organizations dealing with aspects related to rural health. The top websites used in the tweets specialized in neither health care nor public health. The tweets sometimes used wide audience sources, such as international newspapers (The New York Times) or local press. Key opinion leaders have a big influence on the spread of factual information [23], and health authorities could make more use of Twitter to publish news and articles related to rural health and COVID-19.

The most frequently used hashtags were able to uncover interesting and surprising connections to rural health. They included a celebration of the birthday of a top Korean boy band member and the anniversary of the foundation of an Indian political party. These occurred as packed lunches were donated to a rural health center on the front line due to the birthday, and in the case of the political party, it has strong relations with rural areas. The most used hashtag was related to a campaign requesting the appointment of a pharmacist in rural areas, indicating the shortage of pharmacists in these settings. The COVID-19 hashtag was also popular, being used in 2 different forms: “covid19” and “covid.”

The top 10 retweets explicitly mention rural health, health care, or public health problems. The topics are generally of local interest, pointing at very specific issues. Even when rural health is part of a politician’s campaign or a politician’s comment, its interest is local or national. The main uses of Twitter identified in our study are complaints, debates, information sharing, acknowledgements, advertisements, and political campaigns. Regarding the geographical locations of the top tweets, the most influential tweets were derived from India. This is not surprising, given the size of India and the number of rural areas therein. The United States, the Philippines, and South Korea are also among the most frequent locations from where influential tweets were obtained.

The study has several limitations. A circumscribed 57-day time was examined, which may have excluded certain tweets falling outside this period. Another limitation is that the Twitter Search application programming interface can only retrieve data from public-facing Twitter accounts and not from private accounts; however, most accounts are set as public. Another limitation is that as our study retrieved data using a very specific keyword (rural health), our data may have excluded tweets from users who tweeted without using our target keyword. Furthermore, the study retrieved many tweets from other widely spoken languages, such as German or French, which may arise from the limited number of keywords used when retrieving data. Tweets from India occurred in higher frequency than those from other countries. This is potentially because of the huge rural population of the country; this is because India has the largest total rural population [25]. However, our aim was to examine content on Twitter, and content from India happened to be popular at the time we sampled data.

Assessing the needs for those living in rural communities has traditionally been challenging. Several circumstances have been a constraint: language as a barrier, isolation, lack of registries, difficulties to carry out interviews, location of the households, and expenditure to perform studies. Twitter could prove to be a solution for these problems and could be used as a social listening tool to identify the concerns and needs of rural communities. Our study shows that Twitter can be effectively used as a means of communication in rural areas and as a source of information on rural health. Moreover, the information existing on Twitter, when filtered by geographical locations, may be of interest to stakeholders, health care workers, politicians, patients, and communities in general.

Twitter could also be used strategically for those living in rural areas to communicate with one another, for sharing local updates, and to warn of disasters and areas to avoid. It could also be used to connect to share resources and supplies. This could be facilitated using domain-specific hashtags related to each area and widely advertised and popularized locally.

### Conclusions

Twitter has been shown to be a powerful means of communicating about important issues around rural health. Twitter is a tool that can be used to raise awareness of the problems existing in rural health. When examining tweets in English, it was found that India has the most Twitter-related conversations on rural health. Twitter was used to discuss rural settings to express complaints, debate, share information, acknowledge somebody or something, and create advertisements or politician’s campaigns. Twitter could be leveraged as a powerful source of information for individuals and organizations working on rural health and as a means to identify popular narratives and hot issues around this topic.

## Appendix

Multimedia Appendix 1 Time-series chart of Twitter activity.

Multimedia Appendix 2 Associations between word pairs and groups.

## Abbreviations

TAGS: Twitter Archiving Google Sheets
