# Supplementary figures and images for: Analyzing Discussions Around Rural Health on Twitter During the COVID-19 Pandemic: Social Network Analysis of Twitter Data
Source: JMIR Infodemiology. 2023 Mar 8;3:e39209. doi: 10.2196/39209 (PMC10012181; doi:10.2196/39209)

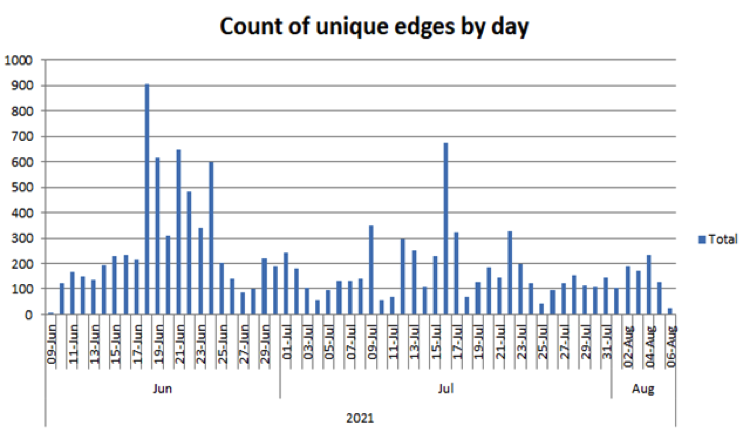

Supplement: Multimedia Appendix 1 [file infodemiology_v3i1e39209_app1.png]
