# Supplementary material for: Analyzing Discussions Around Rural Health on Twitter During the COVID-19 Pandemic: Social Network Analysis of Twitter Data
Source: JMIR Infodemiology. 2023 Mar 8;3:e39209. doi: 10.2196/39209 (PMC10012181; doi:10.2196/39209)
Supplement: Multimedia Appendix 2 [file infodemiology_v3i1e39209_app2.docx]

**Multimedia Appendix 1**

**Table S1.** Word-Pairs Associated with Groups 1

| **Top Word Pairs in Tweet in G1** | **G1 Count** |
| --- | --- |
| rural,health | 874 |
| fighting,covid | 873 |
| covid,question | 873 |
| question,health | 873 |
| health,busting | 873 |
| busting,myths | 873 |
| myths,social | 873 |
| social,media | 873 |
| media,world | 873 |
| world,line | 873 |

**Table S2.** Word-Pairs Associated with Group 2

| **Top Word Pairs in Tweet in G2** | **G2 Count** |
| --- | --- |
| rural,health | 834 |
| health,care | 732 |
| health,systems | 373 |
| expanding,medicaid | 256 |
| taxpayers,money | 254 |
| care,georgia | 216 |
| improve,health | 216 |
| health,outcomes | 216 |
| access,affordable | 212 |
| affordable,health | 212 |

**Table S3.** Word-Pairs Associated with Group 3

| **Top Word Pairs in Tweet in G3** | **G3 Count** |
| --- | --- |
| alan,jones | 1058 |
| rural,health | 684 |
| health,crisis | 618 |
| water,corruption | 560 |
| corruption,rural | 560 |
| friendlyjordies,alan | 529 |
| jones,interviewed | 529 |
| interviewed,times | 529 |
| times,water | 529 |
| crisis,constantly | 529 |

**Table S4.** Word-Pairs Associated with Group 4

| **Top Word Pairs in Tweet in G4** | **G4 Count** |
| --- | --- |
| rural,health | 748 |
| health,care | 131 |
| health,clinics | 89 |
| covid,19 | 80 |
| health,unit | 67 |
| health,clinic | 29 |
| role,rural | 27 |
| increasing,advanced | 25 |
| advanced,practice | 25 |
| practice,providers | 25 |

**Table S5.** Word-Pairs Associated with Group 5

| **Top Word Pairs in Tweet in G5** | **G5 Count** |
| --- | --- |
| rural,health | 871 |
| health,care | 117 |
| sam,rural | 101 |
| health,advocate | 100 |
| health,clinics | 97 |
| health,conference | 72 |
| rural,communities | 53 |
| national,rural | 52 |
| rural,america | 51 |
| covid,19 | 46 |

**Table S6.** Word-Pairs Associated with Group 6

| **Top Word Pairs in Tweet in G6** | **G6 Count** |
| --- | --- |
| य,क | 4176 |
| र,म | 3793 |
| क,न | 2784 |
| न,य | 2784 |
| क,त | 2784 |
| फ,र | 2401 |
| म,स | 2401 |
| स,स | 2401 |
| स,ट | 2401 |
| ट,क | 2401 |

**Table S7.** Word-Pairs Associated with Group 7

| **Top Word Pairs in Tweet in G7** | **G7 Count** |
| --- | --- |
| rural,health | 226 |
| health,centres | 110 |
| primary,health | 69 |
| health,infrastructure | 68 |
| healthcare,system | 58 |
| private,hospitals | 52 |
| state,level | 52 |
| level, organisations | 52 |
| organisations,protest | 52 |
| infrastructure,rural | 52 |

**Table S8.** Word-Pairs Associated with Group 8

| **Top Word Pairs in Tweet in G8** | **G8 Count** |
| --- | --- |
| rural,health | 431 |
| health,care | 425 |
| destroying,rural | 243 |
| attacking,family | 214 |
| family,physicians | 214 |
| physicians,destroying | 214 |
| health,networks | 214 |
| networks,alienating | 214 |
| alienating,nurses | 214 |
| nurses,dismantling | 214 |

**Table S9.** Word-Pairs Associated with Group 9

| **Top Word Pairs in Tweet in G9** | **G9 Count** |
| --- | --- |
| rural,health | 341 |
| happy,birthday | 341 |
| birthday,sunoo | 341 |
| celebration,sunoo's | 339 |
| sunoo's,birthday | 339 |
| birthday,packed | 339 |
| packed,lunchboxes | 339 |
| lunchboxes,frontliners | 339 |
| frontliners,los | 339 |
| los,banos | 339 |

**Table S10.** Word-Pairs Associated with Group 10

| **Top Word Pairs in Tweet in G10** | **G10 Count** |
| --- | --- |
| rural,health | 413 |
| health,care | 65 |
| health,commissioner | 61 |
| health,workforce | 32 |
| national,rural | 32 |
| ruth,stewart | 30 |
| rural,remote | 29 |
| health,brink | 26 |
| brink,workforce | 26 |
| workforce,cliff | 26 |
